# Efficacy analysis of HAIC combined with lenvatinib plus PD-1 inhibitor vs. first-line systemic chemotherapy for advanced intrahepatic cholangiocarcinoma

**DOI:** 10.1038/s41598-024-75102-z

**Published:** 2024-10-14

**Authors:** Zhipeng Lin, Xugong Zou, Xiaolong Hu, Dabei Huang, Yuan Chen, Jiawen Lin, Xiaoqun Li, Jian Zhang

**Affiliations:** Department of Interventonal Medicine, Zhongshan People’s Hospital, Guangdong, 528400 China

**Keywords:** Intrahepatic cholangiocarcinoma, Hepatic arterial infusion chemotherapy, Targeted therapy, Immunotherapy, Cancer, Immunology, Diseases

## Abstract

This research was intended to compare the clinical efficacy of hepatic arterial infusion chemotherapy (HAIC) in conjunction with lenvatinib and PD-1 inhibitors to first-line systemic chemotherapy for advanced intrahepatic cholangiocarcinoma(ICC). The research enrolled advanced ICC patients who underwent HAIC plus lenvatinib and PD-1 inhibitor(*n* = 51) or first-line systemic chemotherapy(cisplatin + gemcitabine, *n* = 39) between July 2020 to January 2023 in Zhongshan People’s Hospital.Their clinical outcomes were assessed through measurement of parameters encompassing objective response rate (ORR), disease control rate (DCR), median overall survival (mOS), median progression-free survival (mPFS), median duration of response (mDOR), and treatment-related adverse events (TRAEs). In accordance with the RECIST1.1, the ORR in the HAIC + L + P and SC groups was 43.1% and 20.5%, while the DCR was 90.2% and 69.2%, respectively (*P* = 0.04 and = 0.02, respectively). The change in the maximum diameter of intrahepatic target lesions in patients before and after treatment and the diameter of intrahepatic tumors in the HAIC + L + P group were sharply smaller versus the SC group ( *P* < 0.001). The HAIC + L + P group had prolonged mOS (16.8 months vs. 11.0 months, *P* = 0.01) and mPFS (12.0 months vs. 6.9 months, *P* < 0.01) in comparison with the SC group. Compared to first-line systemic chemotherapy(cisplatin + gemcitabine), HAIC plus lenvatinib and PD-1 inhibitors contributes to improvement of tumor response and prolongation of OS and PFS in advanced ICC patients.

## Introduction

Cholangiocarcinoma (CCA) is a highly aggressive malignant neoplasm that includes both intrahepatic cholangiocarcinoma(ICC) and extrahepatic cholangiocarcinoma (ECC). ICC represents the world’s second most common primary liver cancer, accounting for 20% of all primary hepatic malignancy. The global incidence of ICC has increased significantly in recent years^1^. Surgical resection is currently considered the primary therapeutic option for ICC removal. However, the majority of patients with ICC are in advanced stages at the time of diagnosis and do not indication for surgery resection^2^. In patients with surgically resected ICC, the recurrence rate can reach up to 60% within two years, with only 9% requiring reoperation and a 5-year survival rate of below 5%^3^. The combination of gemcitabine and cisplatin is commonly used as a primary systemic chemotherapy protocol for advanced ICC, whereas, the mOS rate is only 11.7 months^4^. Gemcitabine combined with oxaliplatin is also a commonly used systemic chemotherapy regimen for advanced ICC, and its effect is comparable to that of gemcitabine combined with cisplatin^5^. With the advancement of medical technology, a variety of methods such as interventional therapy, targeted therapy, immunotherapy, and radiation therapy have begun to be used to treat patients with advanced ICC, and there is need to develop new therapeutic strategies to improve their survival prognosis.

Hepatic arterial infusion chemotherapy (HAIC) involves inserting a catheter using minimally invasive methods into the arterial blood supply of liver tumors, and continuously infusing high-concentration chemotherapy drugs to maximize the destruction of tumor cells. Furthermore, due to the first-pass effect in the liver, the majority of chemotherapy drugs are metabolized with lower systemic exposure, resulting in fewer systemic toxic side effects. Previous research has shown that HAIC can improve survival in ICC patients^6^.

As previously stated, the liver has a first-pass effect on chemotherapy drugs, which results in less systemic exposure after hepatic metabolism. However, in advanced ICC patients, most have extrahepatic lymph node metastases and/or distant metastases, which limits the efficacy of HAIC alone for extrahepatic lesions. Targeted therapy and immunotherapy are promising options for treating extrahepatic lesions. Lenvatinib, a multitarget tyrosine kinase receptor inhibitor, demonstrates potent anti-angiogenic and antiproliferative effects. Previous research has shown that lenvatinib has potent antitumor activity in the treatment of advanced ICC patients^7^. Programmed Death 1 (PD-1), an immune checkpoint blocker, has been shown in previous studies to be effective against advanced ICC^8^. However, the efficacy of targeted or immune therapy alone in advanced ICC patients is unsatisfactory.

HAIC, lenvatinib treatment, and PD-1 inhibitors in antitumor therapy each have distinct therapeutic mechanisms. Consequently, it is hypothesized that the combination of these three treatments will improve survival outcomes of advanced ICC. This retrospectively study paid attention to the clinical efficacy and safety of HAIC + lenvatinib + PD-1 inhibitor combination versus first-line systemic chemotherapy (gemcitabine + cisplatin) for advanced ICC patients.

## Results

### Basic patient information

**Fig. 1:**
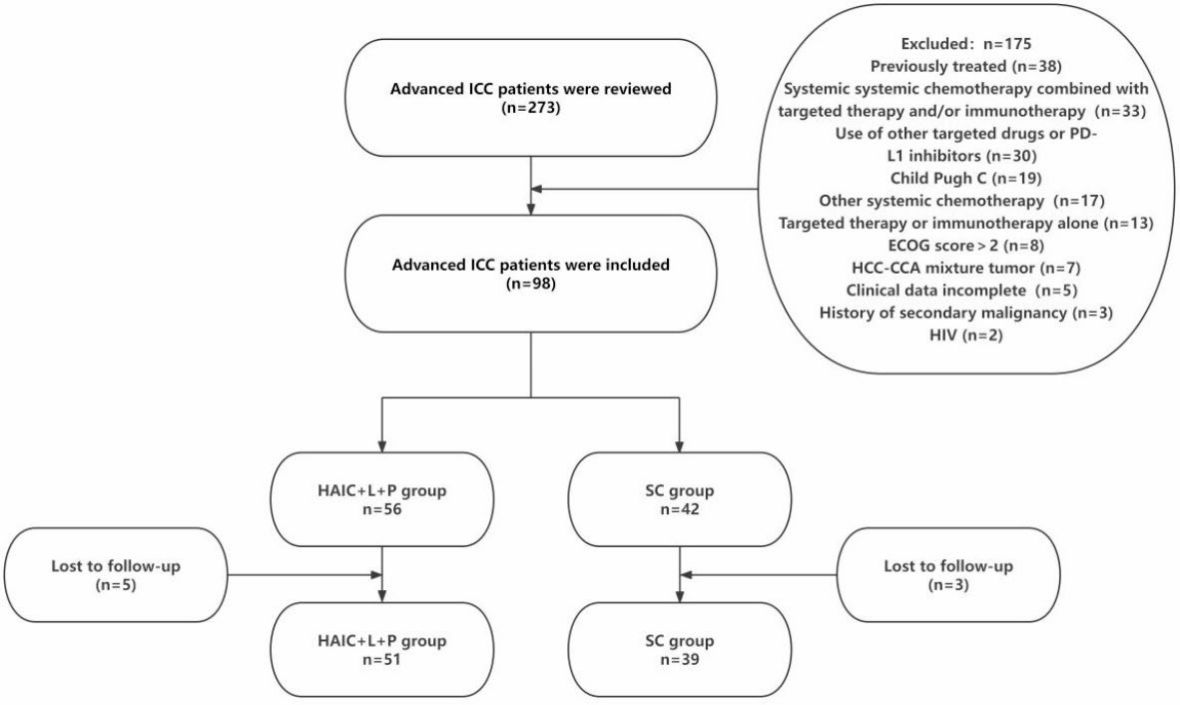
Flow diagram of advanced ICC patients who underwent HAIC combined with lenvatinib plus PD-1 inhibitors or systemic chemotherapy treatment. HCC: hepatocellular carcinoma; CCA: cholangiocarcinoma; ECOG: eastern cooperative oncology group.

The baseline characteristics of both groups are displayed in Table 1.Patients in HAIC + L + P group showed a median follow-up duration of 32.8(23.7, ∞) months with 4.61 ± 2.06 HAIC treatments; while that of patients in the SC group was 22.6 (19.8, ∞) months with 5.51 ± 2.11 systemic chemotherapy treatments (Fig. 1).

**Table 1:** Baseline characteristics.

Characteristics	HAIC + L + *P* Group	SC Group	χ2 Value/ T Value	*P* Value
**Gender**			0.53	0.47
Male	35	23		
Female	16	16		
**Age**				1.00*
< 50	5	4		
≧ 50	46	35		
**HBsAg**			0.84	0.36
Positive	16	8		
Negative	35	31		
**Liver cirrhosis**			0.03	0.87
Yes	11	7		
No	40	32		
**ECOG**			1.08	0.58
0	22	18		
1	23	14		
2	6	7		
**Child Pugh**			0.00	1.00
A	39	29		
B	12	10		
**AFP**	2.9(2.05,4.75)	4.2(2.95,6.00)	10.80	< 0.01
**CA199**	264.1(64.35,806.45)	578.3(213.55,3041.50)	4.63	< 0.01
**CEA**	12.5(2.75,90.60)	41.2(17.50,106.15)	6.26	< 0.01
**Maximum tumor diameter**			1.16	0.56
<5 cm	13	13		
5–10 cm	28	17		
> 10 cm	10	9		
**Number of tumors**			0.96	0.33
≦ 3	22	12		
> 3	29	27		
**PVTT**				0.90*
Vp2	8	10		
Vp3	11	9		
Vp4	2	2		
**Hepatic vein invasion**			0.38	0.54
Yes	8	9		
No	43	30		
**Extrahepatic metastases**			0.17	0.68
Yes	19	12		
No	32	27		
**Lymph node metastases**			0.00	1.00
Yes	43	33		
No	8	6		
**TMN stage**				0.79*
II	5	5		
III	27	22		
IV	19	12		

ECOG: Eastern Cooperative Oncology Group; PVTT: portal vein tumor thrombus; Vp2: the presence of a PVTT in the second-order branches of the portal vein; Vp3: the presence of a PVTT in the first-order branches of the portal vein; Vp4: the presence of a PVTT in the main trunk of the portal vein or a contralateral portal vein branch or both.Labeling with * was calculated using Fisher’s exact probability test.

## Tumor response

**Fig. 2:**
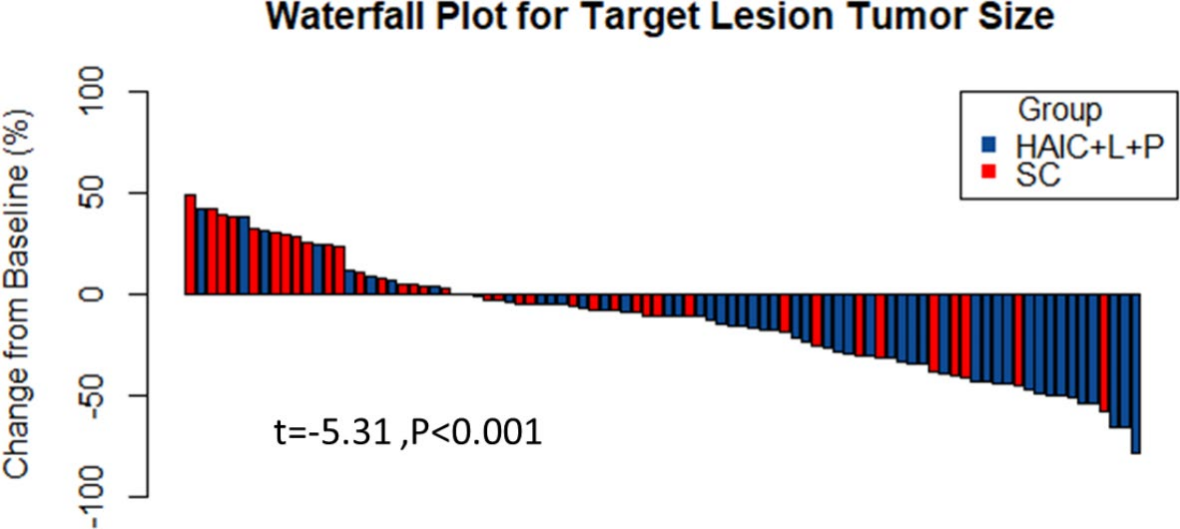
Waterfall plot for tumor size changes in intrahepatic target lesions.

The HAIC + L + P group had 0, 28 (54.9%), 18 (35.3%) and 5 (9.8%) cases of CR, PR, SD, and PD, whereas the SC group had 0, 12 (30.8%), 15 (38.4%) and 12 (30.8%) cases, respectively, when mRECIST was used to assess tumor response. The HAIC + L + P and SC groups had ORR values of 54.9% and 30.8% (*P* = 0.04), and DCR of 90.2% and 69.2% (*P* = 0.02), respectively. The HAIC + L + P group had 0, 22 (43.1%), 24 (47.1%) and 5 (9.8%) cases of CR, PR, SD, and PD, respectively, whereas the SC group had 0, 8 (20.5%), 19 (48.7%) and 12 (30.8%) cases, respectively, when RECIST1.1 was used to assess tumor response. The ORR was 43.1% and 20.5% (*P* = 0.04), while the DCR was 90.2% and 69.2% (*P* = 0.02) in the HAIC + L + P and SC groups, respectively(Table 2).According to the RECIST 1.1 criteria, changes in the maximum diameter of intrahepatic target lesions in patients before and after treatment were compared using waterfall plots, and the diameter of intrahepatic tumors in the HAIC + L + P group was remarkably lower than the SC group(t = -5.31, *P* < 0.001) (Fig. 2).

**Table 2:** Treatment response as assessed by imaging features according to the mRECIST and Recist1.1 criteria in two groups.

Treatment response	mRECIST			RECIST1.1		
	Group.No.(%)			Group.No.(%)		
	HAIC + L + PD-1 group	SC group	P value	HAIC + L + PD-1 group	SC group	P value
CR	0	0	-	0	0	-
PR	28	12	-	22	8	-
SD	18	15	-	24	19	-
PD	5	12	-	5	12	-
ORR,%	28(54.9%)	12(30.8%)	0.04	22(43.1%)	8(20.5%)	0.04
DCR,%	46(90.2%)	27(69.2%)	0.02	46(90.2%)	27(69.2%)	0.02

CR: complete response; PR: partial response; SD: stable disease; PD: progressive disease; ORR: objective response rate; DCR: disease control rate.

## OS, PFS, DOR and factors influencing OS and PFS

**Fig. 3:**
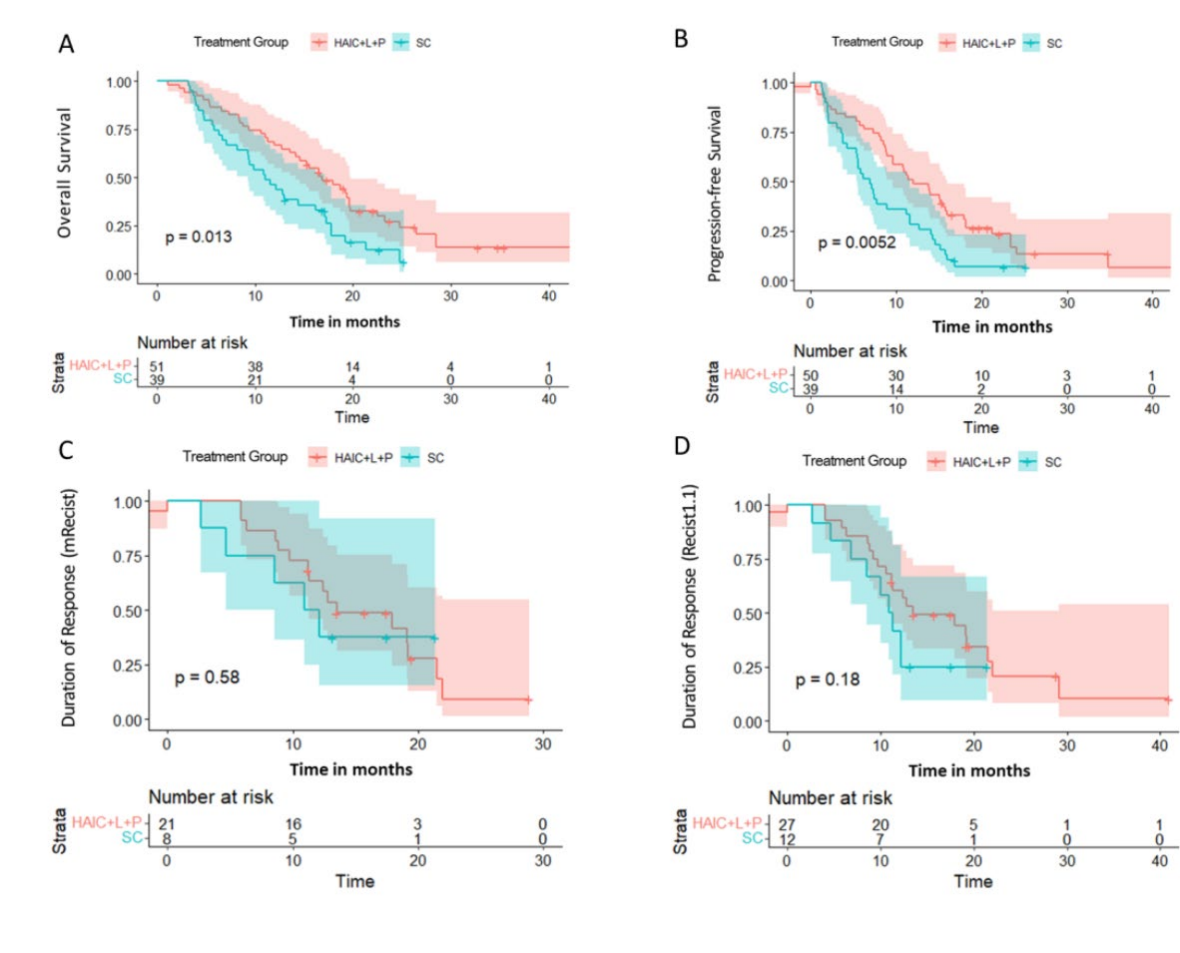
(A) The mOS was 16.8 (95% CI 14.2–22.5) and 11.0 (95% CI 9.3–17.1) months; (B) The mPFS was 12.0 (95% CI 9.6–15.9) and 6.9 (95% CI 5.4–11.5) months; (C) The mDOR (mRECIST) was 13.5 (95% CI 11.3-not reached) and 11.5 (95% CI 8.5-not reached) months. C. The mDOR (RECIST1.1) was 13.5 (95% CI 11.2-not reached) and 11.1 (95% CI 8.5-not reached) months. mOS: mean overall survival; mPFS: median progression-free survival; mDOR: median duration of response.

After follow-up, the mOS values in the HAIC + L + P and SC groups exhibited noticeable differences (16.8 months [95% CI 14.2–22.5] vs. 11.0 months [95% CI 9.3–17.1], χ^2^ = 6.6, *P* = 0.01) (Fig. 3A). The HAIC + L + P group showed sharply longer mPFS (12.0 months [95% CI 9.6–15.9] than the SC group (6.9 months [95% CI 5.4–11.5] (χ^2^ = 7.8, *P* < 0.01) (Fig. 3B). According to mRECIST, the mDOR in the HAIC + L + P and SC groups was 13.5 (95% CI 11.3-not reached) and 11.5 (95% CI 8.5-not reached) months, respectively, with no statistically significant difference (χ^2^=0.3, *P* = 0.58) (Fig. 3C). According to RECIST1.1, the mDOR was 13.5 (95% CI 11.2-not reached) and 11.1 (95% CI 8.5-not reached) months in HAIC + L + P and SC groups, respectively, displaying a statistically insignificant variance (χ^2^ =1.8, *P* = 0.18) (Fig. 3D).

**Fig. 4:**
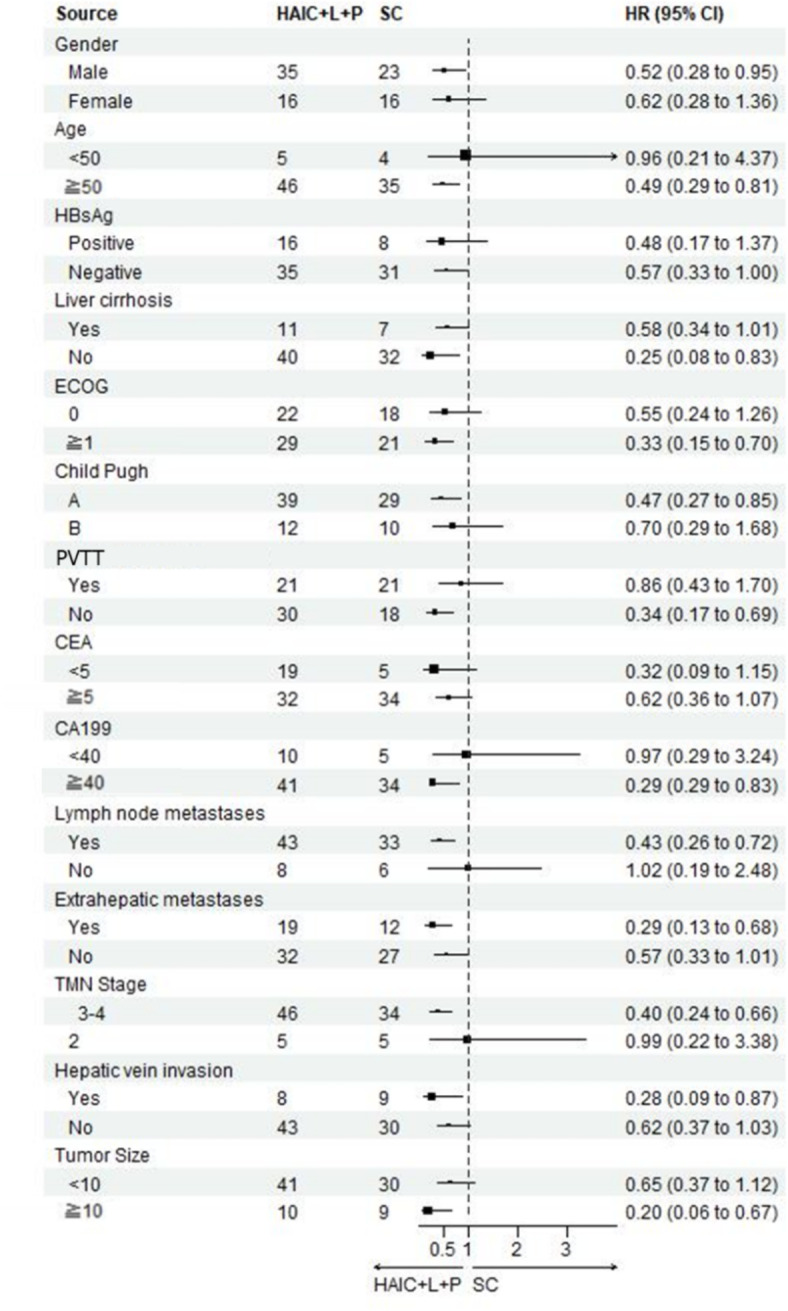
Forest plot of OS for subgroups in patients receiving HAIC combined with lenvatinib plus PD-1 inhibitors or systemic chemotherapy treatment.

It was revealed in the univariate analysis that ECOG ≥ 1 (HR 3.2, 95%CL, 1.94–5.34, *P* < 0.01), Child-Pugh class B (HR 3.2,95%CL, 1.89–5.42, *P* < 0.01), existence of lymph node metastases (HR 3.11, 95%CL, 1.35,7.19, *P* < 0.01), existence of extrahepatic metastasis (HR 2.01, 95%CL, 1.24–3.27, *P* < 0.01), TMN stage 3/4 (HR 3.85, 95%CL, 1.40–10.60, *P* < 0.01), and existence of hepatic vein invasion (HR 1.81, 95%CL 1.04–3.18, *P* < 0.01)were independent risk factors of OS; An analysis of multifactorial COX subgroup is depicted in Fig. 4.

**Fig. 5:**
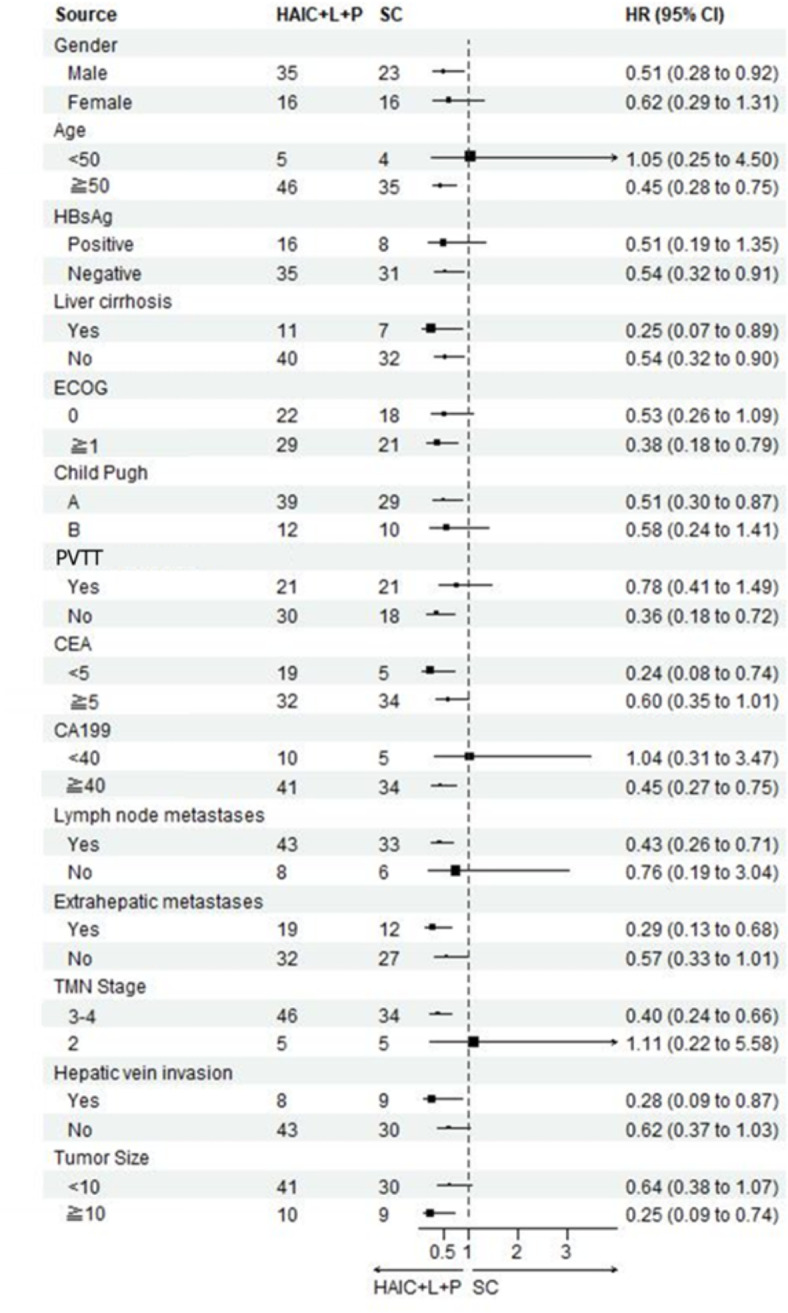
Forest plot of PFS for subgroups in patients receiving HAIC combined with lenvatinib plus PD-1 inhibitors or systemic chemotherapy treatment.

Univariate analysis of factors that impact PFS: existence of cirrhosis (HR 1.87, 95%CL, 1.08–3.23, *P* = 0.02), ECOG ≥ 1 (HR 3.09, 95%CL, 1.88–5.09, *P* < 0.01), Child-Pugh class B (HR 2.76,95%CL, 1.64–4.66, *P* < 0.01), existence of lymph node metastasis (HR 3.49, 95%CL, 1.51,8.08, *P* < 0.01), existence of extrahepatic metastasis (HR 1.82, 95%CL, 1.12–2.96, *P* = 0.01), and TMN stage 3/4 (HR 4.01, 95%CL, 1.46–11.06, *P* < 0.01)were independent risk factors; An analysis of multifactorial COX subgroup analysis is depicted in Fig. 5.

## Treatment safety

A summary of TRAEs is presented in Table 3. No patient experienced treatment-related grade 4 or 5 AEs. Among them, higher levels of ALT and AST(χ^2^=5.69, *P* = 0.02) were prevalent in the HAIC + L + P group. Leukopenia (χ^2^=4.91, *P* = 0.03), thrombocytopenia (χ^2^=6.12, *P* = 0.01), nausea and vomiting (χ^2^=12.52, *P* < 0.01), fatigue(χ^2^=7.49, *P* < 0.01) were greater in the SC group.The lenvatinib dose of 2 patients in the HAIC + L + P group was diminished during the treatment period.No patients discontinued or reduced their use of PD-1 inhibitors during the treatment period.

**Table 3:** Patient treatment-related adverse events.

Adverse Events	Any level of adverse event				Grade 3 adverse events			
	HAIC + L + PD-1 group (*n* = 51)	SC group (*n* = 39)	X^2^ Value	*P* Value	HAIC + L + PD-1 group (*n* = 51)	SC group (*n* = 39)	X^2^ Value	*P* Value
Leukopenia	9(17.6%)	16(41.0%)	4.91	0.03	1(2.0%)	4(10.3%)	-	0.16
Thrombocytopenia	6(11.8%)	14(35.9%)	6.12	0.01	1(2.0%)	4(10.3%)	-	0.16
Rash	6(11.8%)	11(28.2%)	2.90	0.09	0	2(5.1%)	-	0.19
Itchy skin	3(5.9%)	3(7.7%)	-	1.00	0	0	-	-
Hand-foot syndrome	6(11.8%)	2(5.1%)	-	0.46	0	0	-	-
ALT and AST level increase	24(47.%)	8(20.5%)	5.69	0.02	3(5.9%)	0	-	0.26
Serum bilirubin increase	11(21.6%)	3(7.7%)	-	0.09	3(5.9%)	0	-	0.26
Diarrhea	5(9.8%)	3(7.7%)	-	0.72	0	0	-	-
Nausea、Vomit	15(29.4%)	27(69.2%)	12.52	< 0.01	0	5(12.8%)	-	0.01
Proteinuria	8(15.7%)	7(17.9%)	-	0.78	0	0	-	-
Hypothyroidism	8(15.7%)	5(12.8%)	-	0.77	0	0	-	-
Gastrointestinal bleeding	3(5.9%)	1(2.6%)	-	0.07	3(5.9%)	1(2.6%)	-	0.63
Stomach ache	16(31.4%)	6(15.4%)	2.25	0.13	4(7.8%)	1(2.6%)	-	0.38
Weight decreased	7(13.7%)	5(12.8%)	-	1.00	0	0	-	-
Decrease appetite	20(39.2%)	19(48.7%)	0.47	0.49	0	0	-	-
Fatigue	12(23.5%)	21(53.8%)	7.49	< 0.01	0	0	-	-
Elevated creatinine	4(7.8%)	3(7.7%)	-	1.00	1(2.0%)	0	-	1.00
Hypertension	18(35.1%)	0	-	< 0.01	0	0	-	-

## Discussion

Most ICC suffers are diagnosed at later stage, with no indication for surgical intervention, and the disease progresses rapidly with a poor prognosis, severely jeopardizing the health status and life safety of patients.The treatment of advanced ICC patients is difficult, and improving the survival of patients has always been a clinical priority. Gemcitabine plus cisplatin is the primary systemic chemotherapy regimen in the treatment of patients with advanced ICC patients.In a phase III randomized trial(ABC-02 trial), the patients receiving combination therapy with gemcitabine combined plus cisplatin had higher mOS (11.7 months vs. 8.1 months; HR, 0.64; *P* < 0.001), mPFS (8.0 months vs. 5.0 months; HR, 0.63; *P* < 0.001), and DCR(81.4% vs. 71.8%; *P* = 0.049) compared to the patients receiving monotherapy with gemcitabine^4^. Another phase III randomized trial compared the efficacy of gemcitabine plus cisplatin (GC group) and gemcitabine plus tegretol (GS group). The mOS was 13.4 months and 15.1 months in the GC and GS groups, respectively (HR = 0.945; 90% 0.777 ~ 1.149; P for non-inferiority *P* = 0.046). The GS regimen was not inferior to the GC regimen with similar 1-year survival rate(GC vs. GS, 58.3% vs. 59.2%)^9^. There is little difference in survival benefits between different first-line systemic chemotherapy regimens for advanced ICC patients, necessitating the development of new treatment strategies to improve their prognosis. With the ongoing advancement of medical technology, new treatment methods and drugs for advanced ICC are emerging.

The study found notably extended mOS and mPFS and higher ORR (Recist 1.1) and DCR (Recist 1.1 and mRecist) in the HAIC + L + P group in contrast to the SC group. The reduction in intrahepatic tumor diameter was also more pronounced in the HAIC + L + P group. The aforesaid findings indicate that HAIC + L + P treatment has has a greater impact on advanced ICC, improve tumor treatment response in advanced ICC patients, and improves survival prognosis.

HAIC appears to provide distinct advantages in the local treatment of advanced ICC. By precisely inserting a catheter into the hepatic artery, HAIC deliverslarge amounts of chemotherapy drugs directly to liver target lesions. It can treat diffuse lesions, portal vein tumor thrombus, and hepatic vein tumor thrombus, regardless of tumor size or location.The position of the microcatheter in the hepatic artery can be modified based on changes in the lesions during each HAIC treatment. HAIC can concentrate drug concentrations in the liver up to 400 times higher than systemic administration and lower systemic levels of chemotherapy drugs via the liver’s first pass effect, reducing systemic toxicity and adverse reactions^10,11^. A recent prospective phase II study found that the ORR of FOLFOX-HAIC (oxaliplatin plus 5-fluorouracil) treatment for advanced perihilar CCA was 67.6% (25/37), with a DCR of 89.2% (33/37), mPFS of 12.2 months, and OS of 20.5 months^12^. A phase II single-arm study assessed the use of HAIC (floxuridine) in combination with GEMOX chemotherapy, including 38 unresectable ICC patients. The results showed a DCR of 84%, a PR rate of 58%, a mPFS of 11.8 months, a 6-month PFS rate of 84%, and a mOS of 25 months^13^.

The liver first-pass effect, which reduces systemic levels of chemotherapy drugs following HAIC, may result in inadequate control of extrahepatic lesions and progression. Targeted therapy combined with immunotherapy is an effective addition to systemic treatment. Lenvatinib inhibits multiple tyrosine kinase, particularly targeting vascular endothelial growth factor receptor(VEGFR) 1–3, fibroblast growth factor receptor(FGFR) 1–4, RET, KIT, and platelet-derived growth factor receptor β (PDGFRβ)^14^. It is effective in a wide range of solid tumors by inhibiting tumor angiogenesis.Some of the above receptors are highly expressed in ICC, and mature treatment strategies exist for FGFR2 targets. A phase II non-randomized study found that for 107 patients with FGFR2 fusion/rearrangement-positive ICC who progressed after receiving gemcitabine-based chemotherapy, patients treated with the FGFR2 inhibitor Pemigatinib had an ORR of up to 36%, with a CR rate of 3%, PR rate of 33%, mPFS of 6.9 months, and mOS of 21.1 months^15^. A single-arm, multicenter, open-label phase 2 study found that lenvatinib exhibited anti-neoplasm property and tolerable safety in second-line treatment for unresectable biliary tract cancer with an mPFS of 3.19 months and mOS of 7.35 months^7^. Immunotherapy constitutes a promising therapy for various tumors in recent years, and some studies on ICC indicate that immunotherapy may be one of the treatment options for chemotherapy-resistant patients. A retrospective study of 42 patients suffers with advanced ICC intervened with PD-1 inhibitors found a mOS, mPFS, and median time to progression (mTTP) of 19.3, 11.6, and 11.6 months, respectively^8^.

The pathogenesis of ICC has been extensively investigated in recent years, and treatment combinations are expected to improve the clinical efficacy of advanced ICC. In a recent randomized, double-blind, phase III trial (TOPAZ-1), the mOS and mPFS were noticeably different between the gemcitabine + cisplatin + durvalumab and gemcitabine + cisplatin + placebo groups (mOS: 12.8 and 11.5 months, respectively; HR = 0.80; 95% CI, 0.66–0.97; *p* = 0.021; mPFS: 7.2 and 5.7 months, respectively; HR = 0.75; 95% CI, 0.64–0.89; *p* = 0.001). Although the difference in OS was small, the survival curves indicated that up to 25% of patients in the gemcitabine + cisplatin + durvalumab group could survive for more than 2 years, with long-term tumor remission^16^. Another single-center, single-arm, phase II study found that the mOS of toripalimab combination with lenvatinib and GEMOX for advanced ICC was 22.5 months, with an mPFS of 10.2 months, 1- and 2-year OS rates were 76.7% and 49.8%, respectively, and a 1-year PFS rate of 41.4%^17^. In a recent multicenter retrospective analysis, early HAIC incorporated with PD-1 inhibitors significantly prolonged the OS of patients with advanced CCA, with an mOS of 8.8 months and mPFS of 3.7 months^18^.

Since each therapeutic method targets distinct tumor growth pathways, the conjunction of HAIC with lenvatinib and PD-1 inhibitors for treating of advanced ICC has shown promising results. We believe this is related to the following factors: (1) Chemotherapy drugs induce tumor apoptosis through DNA damage and induce immunogenic cell death, enhancing antitumor immune responses and increasing the efficacy of immunotherapy^19^. (2) Small molecule tyrosine kinase inhibitors can improve the microenvironment by VEGFR1-3 and fibroblast growth factor receptor 1 (FGFR1), as well as acting on colony-stimulating factor 1 receptor (CSF1R) to regulate the infiltration of M2-type macrophages infiltration, weaken the immunosuppressive effects of Tregs, enhance the activity of PD-1 and PD-L1 inhibitors, and promote the immune response^20^.

The safety and frequency of adverse reactions are important indicators when evaluating therapy regimens. This study observed that common TRAEs include leukopenia, thrombocytopenia, rash, abdominal pain, nausea, vomiting, decrease appetite, fatigue, elevated ALT levels, elevated AST levels, hypertension, and elevated bilirubin. Overall, the HAIC + L + P group experienced fewer TRAEs than the SC group. The HAIC + L + P group experienced lower episodes of nausea, vomiting, leukopenia, thrombocytopenia, and fatigue, but hypertension and elevated ALT and AST levels were more frequent. The number of grade 3–4 AEs also decreased in this group. The target lesions of ICC are primarily intrahepatic, and HAIC delivers chemotherapy drugs directly to the liver target lesion area via hepatic artery infusion, lowering systemic levels of chemotherapy drugs through the liver first pass effect and reducing systemic toxicity and adverse reactions. In contrast, systemic chemotherapy is administered intravenously, necessitating high levels of drug concentration in the systemic bloodstream, which can harm various organ systems and increase the likelihood of AEs.

Several limitations existed this study. Primarily, treatment regimens were chosen dependent on doctor and patient preferences that generated selection bias into the study cohort. Secondly, the study is a single-center retrospective analysis, which inherently limits the generalizability of the findings. Thirdly, the study relies on a small sample study. Fourthly, the HAIC + L + P group had a relatively short mean follow-up duration. Further research should prioritize prospective, large-sample, randomized controlled trials to validate the findings of this study.

Finally, the treatment combination of HAIC with lenvatinib plus PD-1 inhibitors outperformed first-line systemic chemotherapy in treating advanced ICC. This study improved tumor remission rates, increased OS and PFS, and had a lower incidence of AEs. The results were consistent with expectations for clinical efficacy.

### Methods

#### Patient information

Figure 1 shows a retrospective reviewe of patients treated for advanced ICC at Zhongshan People’s Hospital from July 2020 to January 2023. The study included 90 patients divided into two groups: 51 receiving HAIC plus and PD-1 inhibitor treatment (HAIC + L + P group) and 39 receiving systemic chemotherapy (cisplatin + gemcitabine) treatment (SC group).

Inclusion criteria: (1) Patients without prior ICC treatment who have been diagnosed with advanced ICC by imaging (enhanced MRI and CT) and histopathology; (2) Age > 18 years; (3) Child-Pugh A or B; (4) Eastern Cooperative Oncology Group (ECOG) score of 0–2; (5) Blood counts > 3 × 10^9^/L of white blood cells, absolute neutrophil counts > 1.5 × 10^9^/L of neutrophils, platelet counts > 30 × 10^9^/L of platelets; (6) HAIC + L + P group included patients who chose to give up systemic chemotherapy.

Exclusion criteria: (1) Patients confirmed histologically to have mixed hepatocellular carcinoma and ICC; (2) Infections uncontrolled around the lesion or systemic infections; (3) Symptoms of adverse liver, kidney, cardiac, or pulmonary dysfunction; (4) History of other malignant tumors; (5) Previous infection with human immunodeficiency virus (HIV); (6) For personal reasons, an inability or unwillingness to comply with treatment; (7) Patients with missing clinical data or follow-up.

The Zhongshan People’s Hospital Clinical Research and Application Ethics Committee approved the study, with research project conforming to the *Declaration of Helsinki* as well as ethical principles of research concerning human participants.

## Treatment

The HAIC + L + P group, using a modified Seldinger technique, underwent femoral artery puncture (radial artery/distal radial artery) percutaneously. ① Insert the microcatheter into the corresponding target vessel if the intrahepatic neoplasm has a separate left (LHA) or right hepatic artery (RHA). ② Insert the microcatheter into the appropriate hepatic artery if both LHA and RHA are responsible for tumor’s blood supply. ③ The gastroduodenal artery is embolized with a coil if the intrahepatic tumor is supplied by both LHA and RHA and cannot be avoided gastroduodenal artery, and the microcatheter is placed into the appropriate hepatic artery. The target vessel is completely embolized using embolic material when a small portion of the tumor’s blood supply is found in other arteries (phrenic artery, intercostal artery, etc.).The chemotherapy infusion doses for the gemcitabine and oxaliplatin(GEMOX) regimen include the following: gemcitabine 1000 mg/m2 arterial drip for 2 h on day 1; and oxaliplatin 85 mg/m2 arterial drip for 3 h on day 2. Every 3–4 weeks, the process was repeated. The dose of chemotherapy drugs can be reduced accordingly if the tumor shrinks significantly after treatment.

On the third postoperative day, lenvatinib was administered orally once daily at doses of 8 mg (patients with a weight < 60 kg) or 12 mg (patients with a weight ≥ 60 kg). This dose is reduced to 8 mg/day (if over 60 kg) or 4 mg/day (if under 60 kg) when grade 3 or 4 treatment-related adverse events (TRAEs) occur. PD-1 inhibitors were administered intravenously on the third postoperative day with injections of sintilimab(200 mg) or camrelizumab(200 mg) every 3 weeks. When severe TRAEs occur, corticosteroids are administered. If grade 3 or 4 TRAEs continue, discontinue lenvatinib and/or sintilimab/camrelizumab injections. The dosage may be resumed when toxicity is reduced or the patient can endure this dosage (at the investigator’s discretion).

The SC group received the following treatment: an intravenous drip of cisplatin and gemcitabine. Cisplatin 25 mg/m2 intravenous drip was use on day 1 of each cycle, while gemcitabine 1000 mg/m2 intravenous drip was use on day 1 and 8 respectively. It was repeated every 3–4 weeks. The chemotherapy dosage can be reduced if the tumor shrinks significantly after treatment.

### Data extrection

This study ended on January 31, 2024. Each subsequent appointment included a review of medical history, a physical exam, an enhanced abdominal CT/MRI scan, standard blood tests, standard routine urine, standard stool tests, hepatic and renal function assessments, coagulation function tests, thyroid function tests, blood glucose testing and detection of tumor markers. CT or MRI was utilized to scan corresponding extrahepatic metastatic loci.

### Efficacy and safety evaluation

The study’s primary focus was on OS, with secondary outcomes including progression-free survival (PFS), duration of response (DOR), objective response rate (ORR), disease control rate (DCR), and treatment-related adverse events (TRAEs). Tumor response was assessed independently *via* two experienced diagnostic imaging doctors, both having over 10 years of experience. Consensus was reached through a discussion of the RECIST 1.1 and mRECIST criteria for assessing tumor response: complete (CR) or partial response (PR), stable disease (SD), and progressive disease(PD)^21,22^. ORR was calculated as the percentage of participants with a CR/PR. DCR represented the percentage of participants who did not have PD. PFS denoted the time from the beginning to the first instance of disease progression or death (whichever happened first). OS signified the time between the initiation of treatment and the occurrence of death for any reason. DOR was defined as the time from the initial diagnosis of CR/PR to the onset of progression or death (whichever happened first). During patient follow-up, TRAEs were assessed following CTCAE version 5.0 and recorded^23^.

### Statistical analysis

Statistical analysis was performed using SPSS 20.0 and R 4.3.1, with normally distributed data summarized in the form of means ± standard deviations and skewed data presented as medians (interquartile ranges). Count data were presented as frequencies. The χ^2^ test or Fisher exact probability test was employed for the baseline data comparison of the HAIC + L + P and SC groups. OS, PFS, and DOR in two groups were compared by the Kaplan–Meier log-rank test. Changes in intrahepatic tumor diameter before and after treatment in two groups were compared by the independent samples t-test. Analysis of the possible influencing factors for OS and PFS was conducted using Cox’s proportional hazards regression. Variables showing *P* < 0.10 in the uni-variable analysis were selected in the multi-variable analysis. The COX subgroup analysis results were visualized in forest plots. The level of statistical significance was set at *P* < 0.05.

## Data Availability

The datasets used and/or analysed during the current study available from the corresponding author on reasonable request.
